# Brief report on ecological momentary assessment: everyday states predict HIV prevention behaviors

**DOI:** 10.1186/s13104-015-1814-4

**Published:** 2016-01-04

**Authors:** Paul F. Cook, Catherine J. McElwain, Lucy A. Bradley-Springer

**Affiliations:** University of Colorado College of Nursing, Campus Box C288-04, Aurora, CO 80045 USA; University of Colorado School of Medicine, 12631 E. 17th Ave., Mail Stop 8204, Aurora, CO 80045 USA

**Keywords:** Ecological momentary assessment, HIV, Motivation, Prevention, Theory

## Abstract

**Background:**

Prevention behaviors help persons living with HIV (PLWH) to avoid transmitting HIV, and psychological variables have been found to predict HIV prevention behaviors. These variables have typically been measured using retrospective questionnaires about average psychological states over a period of time, which are likely to be biased by selective recall and interpretation. Measuring the same variables as momentary states, in the day-to-day context where they actually occur, may reveal different relationships to behavior.

**Findings:**

21 PLWH completed daily surveys about momentary states and prevention behaviors. Brief, validated measures were used to assess control beliefs, mood, stress, coping, social support, stigma, knowledge, and motivation. We used multilevel models to predict prevention behaviors from momentary states the previous day, while controlling for the effect of multiple observations from the same person over time. Participants reported a moderate overall level of HIV prevention behaviors during the 6-month study. Although lapses in prevention were infrequent, there was room for improvement. Control beliefs, mood, and motivation had significant prospective effects on HIV prevention behaviors, *r*s = 0.07−0.21. Stress and coping had effects approaching significance.

**Conclusions:**

Some momentary states predicted prevention behaviors, providing partial support for the motivational model. This finding supports past research showing effects of momentary states on behavior, and advances the science by testing multiple predictors. High within-sample diversity strengthened generalizability, but the overall sample size was small and the findings require replication. Future research should continue to examine the everyday experiences of PLWH as influences on their behavior.

## Findings

### Background

Preventing transmission of human immunodeficiency virus (HIV) is a major public health issue. Behaviors like abstinence, condom use, and/or using clean needles help persons living with HIV (PLWH) avoid transmitting HIV to others [1]. Although many PLWH take precautions to avoid transmitting HIV, a significant number engage in behaviors that place others at risk: for instance, 26 % of anal sex occurrences for HIV-positive men who have sex with men (MSM) are unprotected even when their partner is HIV-seronegative or of unknown serostatus [2]. In addition to reducing new infection rates, prevention using condoms benefits PLWH by reducing their chance of acquiring other sexually transmitted diseases or treatment-resistant strains of HIV.

Although prevention programs for PLWH have been developed based on various theoretical models and many of these are efficacious [3], the predictors of PLWH’s HIV prevention behaviors are still not well understood. One meta-analysis found that the strongest predictors of prevention behaviors among PLWH were belief in one’s control over situations and behaviors, positive mood, having information about HIV, being concerned about HIV, social support, ability to communicate about HIV, motivation to have safer sex, lack of perceived barriers to condom use, absence of avoidant coping strategies, and absence of psychotic symptoms or sexual compulsiveness [4].

#### Momentary states as predictors of HIV prevention behavior

Many health phenomena including HIV prevention behaviors can be meaningfully understood as *momentary states* that have high levels of within-person variability and fluctuate during everyday life [5–7]. However, most research [4] has measured variables retrospectively and in aggregate as *traits*, which are long-term and stable patterns of interpreting and responding to events. For example, researchers might ask PLWH about their typical mood or usual motivation over a period of days or weeks, then correlate these reports with similar aggregate measures of behavior. Studies have shown only moderate correlations between state and trait metrics of the same construct [8], and state- versus trait-level measures of the same construct may predict behavior in different ways. This discrepancy results from cognitive biases in recall and interpretation, which link trait-level aggregate measures more closely to people’s ideas about themselves than to their actual experiences in the moment [9].

Ecological momentary assessment (EMA) methods evaluate momentary states and behaviors close to the time they actually occur [10], and can reveal new insights about the predictors of HIV prevention behaviors. A few studies using EMA suggest that HIV prevention behaviors depend on momentary mood [11, 12] and contextual factors including alcohol use [13, 14]. Mustanski’s study [12] included a specific comparison of the same variables measured as momentary states and as stable traits, and found that state-level emotion measures—in particular high anxiety and low positive affect—had stronger effects than trait-level emotion measures on HIV prevention behaviors.

#### Theoretical model and goals of the current study

This pilot study examined potential momentary state predictors of HIV prevention behaviors based on a motivational model in which five momentary state variables predict motivation for HIV prevention, which in turn predicts HIV prevention behaviors [15]. The model was derived from leading theories and research on trait-level predictor variables: *control beliefs* and *social support* as in the Theory of Planned Behavior [TPB] [16]; *motivation* as in the Information-Motivation-Behavioral Skills [IMB] Model [17]; *stress*, *coping*, and *mood* as in Leventhal’s dual-process model of health behavior [18]. We also tested *HIV stigma* as an additional variable inversely related to social support, and *information* as a variable that some authors have considered important (as in the IMB model) but that others have found to be unrelated to behavior at the trait level (as in the TPB). Crepaz and Marks’s meta-analysis [4] also found trait-level effects for information, control beliefs, mood, social support, coping and motivation on prevention.

Given that a single failure of HIV prevention behaviors can lead to transmission, and that these behaviors occur relatively infrequently in the context of everyday life, the future development of both theory and interventions depends on a better understanding of state-level predictors of behavior. Overall patterns tend to be easier to discern than the variables that predict specific incidents, but they are less useful when attempting to intervene [19]. Although many of the predictor variables included in this pilot study are well supported by prior research, the element of *time* in state-level analyses introduces additional within-person variability and limits the applicability of trait-level theories in predicting state-level behaviors [20]. Our goal in the current study was therefore to screen multiple predictors suggested by the motivational model of momentary state influences on behavior [15], in order to guide further theory development and research.

### Methods

Data were collected in a pilot study using daily electronic surveys with PLWH. The study’s methods are published elsewhere [15], but summarized below.

#### Participants

This study was conducted in accordance with the Declaration of Helsinki and approved by the Colorado Multiple Institutional Review Board (protocol #06-0948). Participants were 21 PLWH recruited from an outpatient infectious disease practice in Denver, CO. Another 15 PLWH invited to the study declined to participate, primarily because of the time commitment required. Quota sampling was used to recruit a diverse sample in terms of race/ethnicity and gender, approximating the current demographics of the U. S. HIV epidemic [15]. Participants’ demographic characteristics are summarized in Table 1. Attrition was 33 % over 6 months, with some evidence that minority PLWH were more likely to refuse participation and also to leave the study early. Detailed recruitment, participation, and attrition data are published elsewhere [15].

**Table 1:** Participant demographics (N = 21)

Characteristic	M (SD) or frequency (%)
Age	42.0 years (8.8 years)
Gender	15 men (71 %) 6 women (29 %)
Race/ethnicity	9 African–American (43 %) 7 White non-Hispanic (33 %) 5 White Hispanic (24 %)
Sexual orientation	14 MSM (67 %) 1 heterosexual man (5 %) 6 heterosexual women (28 %)
Injection drug use	3 injection drug users (14 %) 18 non-IDU (86 %)

*MSM* men who have sex with men, *IDU* injection drug user

#### Procedure

Each participant was given a personal digital assistant (PDA) handheld computer and asked to complete a 5-minute questionnaire (26–46 questions, depending on skip logic) once daily at a time they had pre-selected. Participants completed the questionnaire once at the start of the study with a research assistant, to ensure that they understood the questions and the response choices. PDAs were password-protected. Daily responses were date- and time-stamped. Participants completed questionnaires on 72 % of study days (range 37–92 %) for an average of 4 months (range 1–8). A total of 2319 daily questionnaires were completed by 21 PLWH during the 6-month study. The original study was designed to identify the optimal duration of daily monitoring, so variability in the number of data points per individual was expected. PLWH returned to the clinic in person to download data from their PDA every 2 months. At these visits, PLWH also answered open-ended questions to identify any additional factors that they believed impacted their HIV prevention behaviors. Participants were paid $25 per visit, and were allowed to keep their PDAs if they participated for at least 2 months.

#### Measures

Daily PDA questionnaires were used to evaluate participants’ state-level control beliefs,^1^ mood, perceived social support, experience of stigma related to HIV, information received about HIV, stress experienced, coping strategies used, and motivation for HIV prevention. The *stress* scale had separate items for acute and chronic stressors. The *coping* scale included a multi-item scale on specific coping strategies used, plus a separate single item about whether the selected coping strategies successfully resolved the problem. The *information* scale was coded *yes/no* to correct for significant skew. *Motivation* was measured using a proxy variable, based on participants’ positive or negative evaluation of any new information received about HIV prevention. As a check on the comprehensiveness of the list of potential predictors, we asked participants at the end of the study to suggest any other important predictors of their HIV prevention behaviors that we did not consider.

For the dependent variable in the analysis, participants answered questions about their HIV *prevention behaviors* (e.g., carrying condoms, avoiding risky situations). Items were taken from the Behavioral Risk Factor Screening Survey, a measure of prevention behaviors developed by the Centers for Disease Control and Prevention (CDC) and used for population-level epidemiological data collection [21]. To improve reliability of the CDC’s original *yes/no* items, our 7-item measure substituted response choices from a well-validated tool for daily survey data collection, the Diary of Ambulatory Behavioral States [22], that asked participants to rate their performance of each behavior on a 4-point scale (1 = *NO!!*, 2 = *no??*, 3 = *yes??*, 4 = *YES!!*). In a prior study, participants said that these response choices were easy to use and to understand, with the lower-case font and question marks indicating less certainty than the capital font and exclamation points, and items using this response scale showed good reliability [15]. Because multiple prevention strategies may be used, any prevention is better than none, and using more strategies may be more effective, an average of the seven prevention items was used for analysis. Other items asked about HIV risk behaviors (e.g., unprotected sex, sharing needles), but these had too little variability for analysis because most PLWH reported no risk behaviors. The risk items also appeared to be more vulnerable to social desirability bias than the prevention items. Most measures were non-reactive with repeated use and all had adequate reliability. Additional measurement detail is provided in [15].

#### Data analysis

Data were analyzed using a prospective prediction strategy in which each participant’s scores on each momentary state predictor variable were compared with their self-reported behavior at the subsequent data collection point, which was usually the next day. Multilevel models were used to correct for the intra-class correlation (*ICC*) of data points from the same participant, which in this study was high for most variables, *ICC*s = 0.64–0.93 [15]. Nonparametric multilevel models were used to correct for skew in the dependent variable [23]. In tests of within-person relationships, power for multilevel models is based on the number of observations adjusted for the *ICC*, rather than on the number of participants. With an average of 110 data points from each of 21 participants, power was .80 to detect moderate effects of *r* = 0.48 or larger at *α* = 0.05 [24]. We first tested for the effect of time on prevention behaviors, then tested each potential predictor variable individually. Alpha was not corrected across models in this preliminary study.

### Results

#### HIV prevention behaviors

Participants reported an overall moderate level of HIV prevention behaviors during the study, *M* = 2.52 out of 4 possible points (*SD* = 0.45). The range of participant responses was slightly restricted, 1.22–3.11 on a 1–4 scale, meaning that no participant ever said they used *no* prevention strategies, and no participant ever endorsed *all* prevention items over a 6-month monitoring period. There was some negative skew in the prevention measure, skew/*SE*_*skew*_ = −2.63, meaning that participants gave higher scores on the prevention scale more often than they gave lower ones. We corrected for skew using nonparametric analyses. A multilevel model with time as a level-1 predictor and data clustered within individuals showed that individual participants’ prevention behaviors were relatively stable based on a flat linear trend, *t*(8) = −0.25, *p* = 0.81, *β* = −0.0001. Although relatively high ICCs for all momentary state variables mean that these states fluctuated only slightly over time, the ICC for prevention behavior was particularly stable at .93. This suggests that gaps in HIV prevention behaviors are relatively infrequent and therefore potentially hard to predict.

In an interview question asked during the baseline assessment, 100 % of participants said they had adequate knowledge of how to prevent HIV transmission. Interestingly, 70 % (16/21) said they also had adequate knowledge of how to prevent HIV infection at the time they were originally infected with HIV. Neither current nor past self-reported knowledge of HIV prevention had any relationship to current HIV prevention behaviors as reported on the daily questionnaires, *p*s > 0.58. Furthermore, there were no significant relationships between HIV prevention behaviors and any of PLWH’s momentary states measured on the *same* day, all *p*s > 0.05.

#### Prediction of HIV prevention behaviors from momentary states

Table 2 shows the relationship of each momentary state variable to PLWH’s self-reported HIV prevention behaviors at the next daily data collection point. Scores on the prevention behaviors scale were significantly related to participants’ prior-day report of higher control beliefs, *r* = 0.10, *p* < 0.001, better mood, *r* = 0.07, *p* = 0.004, and higher motivation based on a positive evaluation of new information received, *r* = 0.21, *p* = 0.05. Chronic stress, *r* = 0.07, *p* = 0.13, and successful coping, *r* = 0.06, *p* = 0.19, did not reach conventional levels for statistical significance as predictors of prevention behaviors, and their effect sizes were small. Furthermore, neither acute stress nor the number of coping strategies used predicted HIV prevention behaviors. Additionally, HIV-related stigma, social support, and information each failed to prospectively predict HIV prevention behaviors.

**Table 2:** Predictors of PLWH’s self-reported HIV prevention behaviors

Construct	Effect on next-day prevention scale score
Control beliefs^a^	*T* (1630) = 3.95, *p* < 0.001, *r* = 0.10***
Mood	*T* (1639) = 2.96, *p* = 0.004, *r* = 0.07**
Stigma	HIV stigma scale: *T* (1618) = −0.84, *p* = 0.40, *r* = 0.02
Stress	Acute stress item: *T* (1622) = 0.08, *p* = 0.94, *r* < 0.01
	Chronic stress item: *T* (466) = −1.52, *p* = 0.13, *r* = 0.07
Coping	Assessment of daily coping: *T* (231) = 0.86, *p* = 0.39, *r* = 0.06
	Successful resolution item: *T* (466) = −1.30, *p* = 0.19, *r* = 0.06
Social support	*T* (1631) = −0.57, *p* = 0.57, *r* = 0.01
HIV stigma	*T* (1631) = 0.06, *p* = 0.95, *r* < 0.01
Information source	*T* (85) = 0.80, *p* = 0.43, *r* = 0.09
Motivation^a^	*T* (85) = 1.97, *p* = 0.05, *r* = 0.21*
Risk behaviors	*T* (1612) = 0.55, *p* = 0.58, *r* = 0.01

* *p* ≤ 0.05, ** *p* ≤ 0.01, *** *p* ≤ 0.001

^a^Variable names clarified from Cook, et al., 2010: Control Beliefs were originally labeled “Negative Thoughts” which sounds misleadingly like Mood; and in the current study Motivation is a proxy variable as described in the text, labeled “Evaluation of Source” in the 2010 paper

#### Additional possible predictors identified by participants

In addition to the momentary state variables included in daily surveys, participants said that health context variables were important for their HIV prevention behaviors. For instance, participants said they were more likely to take steps for prevention when they were feeling well, but that they also might be more likely to encounter risky situations. Participants suggested that the researchers should ask further questions about medication use, side effects, and stigma experiences with health care providers, especially new providers. Despite a lack of significant findings on the coping scale, participants’ feedback indicated that this set of items was interesting and relevant to their daily lives: some participants stated that the questionnaire made them more aware of how stress impacted their daily moods and reactions, while others said it made them more aware of their own coping strategies.

### Discussion

PLWH reported relatively high and consistent prevention behaviors over the course of this study, and these did not correlate with momentary states measured on the same day. However, momentary states did prospectively predict HIV prevention behaviors the next day. Consistent with research on trait-level predictors of HIV prevention behaviors [25], this included significant effects for control beliefs, mood, and motivation. Consistent with theory [15] and prior research [25], new information did not predict prevention behaviors. Contrary to theory [15], neither social support nor stigma predicted prevention behaviors; this was contrary to past research at the trait level showing a significant effect of social support [25]. Stress and coping were each measured with two subscales, which showed nonsignificant effects. These predictor variables have each been found significant at the trait level [25], and their failure to predict behaviors in the current study may have been due to sample size or measurement limitations. Based on these considerations, the relevance of stress and coping at the momentary state level remains unclear.

The current study confirms previous findings about the state-level effects of mood [12], supports other state-level predictors that have previously been tested only as traits, and advances the science of momentary state influences on behavior by testing multiple predictors in an integrated theory of state-level influences on behavior. Prior studies have examined only one or a few state-level predictors and had limited theoretical grounding. Although not all variables in the theoretical model underlying this study were confirmed to predict HIV prevention behaviors, the model received partial support.

The current study tested whether momentary states *prospectively* predicted HIV prevention behaviors on the next day, which is a slightly different strategy from many EMA studies that look at relationships between momentary states and behaviors measured concurrently—e.g., [26]. Even though the next day’s prevention behaviors were more distant in time from the assessment of momentary states, our primary objective was to establish potential causation between predictor variables and prevention behaviors when both were measured as state-level constructs, a goal that was supported by the prospective daily data analysis strategy employed. Additionally, most prevention behaviors are actions taken prospectively (e.g., carrying condoms), so it may be most useful to focus on variables that predict next-day prevention behaviors as early warning signs that can be used to direct needed interventions before lapses in prevention occur.

#### Limitations and directions for future research

Because the current findings are based on a small sample, they may not generalize to all PLWH, and even predictors with weak effects might still be appropriate for further research. Although this study used quota sampling to recruit PLWH who were demographically similar to the U.S. HIV epidemic as a whole, results still may generalize primarily to White PLWH because of the higher attrition among minority PLWH. All data provided by minority participants were included in the analysis, but the total number of data points from this group was smaller. Statistical power was adequate to detect moderate effects based on over 2300 daily questionnaires completed by participants, but high between-participant variability in a small sample might have contributed to weaker than expected effects. The number of participants is comparable to the small *N* in some prior EMA studies of PLWH [27], although at least two larger-scale studies have been conducted on individual risk factors: alcohol [13] and mood [12]. Statistical models with multiple simultaneous predictors were not used in the current analysis because of the high risk of sample-dependent results and a potential lack of generalizability when data-driven decision rules are used with a small sample. Future research should (a) include a larger number of participants; (b) collect many daily data points per participant to capture state-level variability; (c) include multiple predictors of prevention behaviors in a single multiple-variable statistical model to test their relative importance; and (d) test predictors such as alcohol and mood that are well supported by prior research as well as those identified as potentially meaningful in small-scale pilot work such as the current study.

To partially address the limitations of significance testing with a small sample, we reported effect sizes (correlation coefficients) for all analyses to quantify the relative importance of each studied predictor variable. Additionally, we asked participants open-ended questions to identify other variables that might be important to study. These questions revealed that PLWH saw healthcare variables including adherence, side effects, and provider relationships as potentially important for future research of this type. In addition, PLWH emphasized the importance of coping as an important factor in HIV prevention behaviors; even though this study showed small and nonsignificant effects of stress and coping on prevention behaviors, based on the findings of other studies [4] and participants’ suggestions in the current study these may still be potentially important variables for future research. Because the receipt of new information showed no relationship to prevention, but motivation for prevention after receiving new information was a better predictor, future studies should examine motivation separately from whether participants received any new information about HIV.

### Conclusions

Momentary states are under-studied, proximal variables that appear to predict HIV prevention behaviors among PLWH, and that may have different effects from trait-level predictors because they are less affected by biases associated with recall and interpretation. Despite a small sample size, our study gathered a large amount of data from individual participants and examined a range of predictor variables in order to enhance the limited literature on momentary state predictors of PLWH’s prevention behaviors. Further, we used a prospective analysis strategy examining the effects of momentary states on behavior at the next daily data collection point, a novel method that strengthens causal interpretations. Additional research using EMA is needed to clarify the momentary states that are most conducive to HIV prevention behaviors.

## Abbreviations

EMA: ecological momentary assessment
HIV: human immunodeficiency virus
ICC: intra-class correlation coefficient
IDU: injection drug user
IMB: information-motivation-behavioral skills model
MSM: men who have sex with men
PDA: personal digital assistant device
PLWH: persons living with HIV
TPB: theory of planned behavior

## Footnotes

1 Although this variable was labeled “negative thoughts” in our initial methods paper, it is more accurately described as “control beliefs” based on item content. This label is also more useful for placing current findings in the context of prior research.
